# ALS-linked FUS mutations confer loss and gain of function in the nucleus by promoting excessive formation of dysfunctional paraspeckles

**DOI:** 10.1186/s40478-019-0658-x

**Published:** 2019-01-14

**Authors:** Haiyan An, Lucy Skelt, Antonietta Notaro, J. Robin Highley, Archa H. Fox, Vincenzo La Bella, Vladimir L. Buchman, Tatyana A. Shelkovnikova

**Affiliations:** 1 School of Biosciences, Cardiff University, Sir Martin Evans Building, Museum Avenue, Cardiff, CF10 3AX UK; 2 ALS Clinical Research Center and Laboratory of Neurochemistry, Department of Experimental Biomedicine and Clinical Neurosciences, University of Palermo, Palermo, Italy; 3 The Sheffield Institute for Translational Neuroscience, Sheffield, S10 2HQ UK; 4 School of Human Sciences, School of Molecular Sciences and Harry Perkins Institute of Medical Research, University of Western Australia, Crawley, 6009 Australia; 5 Institute of Physiologically Active Compounds RAS, Chernogolovka, Russian Federation 142432; 6 Medicines Discovery Institute, Cardiff University, Cardiff, CF10 3AT UK

**Keywords:** Amyotrophic lateral sclerosis (ALS), Fused in sarcoma (FUS), NEAT1, Paraspeckle

## Abstract

Mutations in the *FUS* gene cause amyotrophic lateral sclerosis (ALS-FUS). Mutant FUS is known to confer cytoplasmic gain of function but its effects in the nucleus are less understood. FUS is an essential component of paraspeckles, subnuclear bodies assembled on a lncRNA NEAT1. Paraspeckles may play a protective role specifically in degenerating spinal motor neurons. However it is still unknown how endogenous levels of mutant FUS would affect NEAT1/paraspeckles. Using novel cell lines with the *FUS* gene modified by CRISPR/Cas9 and human patient fibroblasts, we found that endogenous levels of mutant FUS cause accumulation of NEAT1 isoforms and paraspeckles. However, despite only mild cytoplasmic mislocalisation of FUS, paraspeckle integrity is compromised in these cells, as confirmed by reduced interaction of mutant FUS with core paraspeckle proteins NONO and SFPQ and increased NEAT1 extractability. This results in NEAT1 localisation outside paraspeckles, especially prominent under conditions of paraspeckle-inducing stress. Consistently, paraspeckle-dependent microRNA production, a readout for functionality of paraspeckles, is impaired in cells expressing mutant FUS. In line with the cellular data, we observed paraspeckle hyper-assembly in spinal neurons of ALS-FUS patients. Therefore, despite largely preserving its nuclear localisation, mutant FUS leads to loss (dysfunctional paraspeckles) and gain (excess of free NEAT1) of function in the nucleus. Perturbed fine structure and functionality of paraspeckles accompanied by accumulation of non-paraspeckle NEAT1 may contribute to the disease severity in ALS-FUS.

**Electronic supplementary material:**

The online version of this article (10.1186/s40478-019-0658-x) contains supplementary material, which is available to authorized users.

## Introduction

Amyotrophic lateral sclerosis (ALS) is a severe adult-onset neurodegenerative disease affecting motor neurons. More than 20 genes have been linked to familial (f)ALS, and many of them encode RNA-binding proteins, including FUS [61]. Over 50 mutations in the *FUS* gene have been found in fALS and sporadic (s)ALS patients, the vast majority being heterozygous mutations with autosomal dominant inheritance; most of them affect the nuclear localization signal (NLS) of the protein [31, 33, 34, 65]. Mutations in the *FUS* gene cause an aggressive, sometimes juvenile-onset disease [34].

The histopathological hallmark of ALS-FUS is partial mislocalisation of this predominantly nuclear protein to the cytoplasm in neurons and glial cells of the spinal cord and formation of FUS-positive inclusions [23, 31, 65]. It should be noted, however, that significant FUS mislocalisation is seen only in a subset of ALS-FUS cases and only in a subset of neurons in the latter cohort [23, 29, 39], suggesting that altered nuclear function(s) of mutant FUS can drive pathological changes sufficient to cause the disease. Indeed, FUS mutations having only a minor effect on its nuclear import, such as R521G(H), are detrimental in in vitro and in vivo models [47, 49, 51, 66]. In addition, ALS-linked FUS mutations outside its NLS have been identified [64], and they also cause pathological cellular phenotypes [45, 46]. Finally, most recent studies from mouse models of FUS pathology revealed that mutant FUS is able to cause neurodegeneration in the absence of cytoplasmic pathology and even significant mislocalisation, strongly suggesting that nuclear gain of toxic function by mutant FUS represents an important disease mechanism [15, 37]. Despite significant progress in our understanding of cytoplasmic gain of function by mutant FUS [19], nuclear mechanisms of mutant FUS toxicity are still poorly understood.

Paraspeckles are RNA granules formed in the nuclear interchromatin space, in close proximity to splicing speckles [20]. Paraspeckles contain several core and multiple secondary proteins that are assembled on a scaffold long non-coding RNA (lncRNA) NEAT1 [12, 42, 50, 58]. The *NEAT1* gene produces two transcripts, NEAT1_1 and NEAT1_2; only the longer isoform, NEAT1_2, is capable of forming paraspeckles [42]. Established functions of paraspeckles include sequestration of some RNAs and transcription factors, and thus regulation of gene expression, in response to certain stimuli such as proteasomal inhibition and viral infection [1, 10, 24, 26, 73]. Most recently, roles for paraspeckles in enhancing microRNA biogenesis and regulation of mitochondrial function have been identified [27, 67]. Dysfunction of paraspeckles or their components is implicated in the increasing number of human diseases, including cancer, autoimmune and neurodegenerative disorders [21].

FUS is involved in multiple processes related to cellular RNA metabolism [48]. The protein possesses a low-complexity prion-like domain responsible for its ability to phase-separate and to be recruited into RNA granules in the nucleus or cytoplasm [6, 56]. Although normal and mutant FUS are incorporated into a variety of RNA granules and can even nucleate RNA granules when accumulated [3, 18, 54, 72], the paraspeckle is the only type of physiological RNA granule which requires FUS as a structural component. FUS is defined as an essential paraspeckle protein, in that its knockdown eliminates paraspeckles [42, 55].

Paraspeckles likely play an important role in ALS pathogenesis. Indeed, paraspeckle proteins are enriched in the pool of proteins affected by ALS-causative mutations [2]. Although healthy mammalian neurons lack NEAT1_2 expression and hence paraspeckles in vitro and in vivo [43, 53], de novo paraspeckle formation is typical for spinal motor neurons of sALS and fALS patients and as such can be considered a hallmark of the disease [44, 53]. Previously, we reported pathological aggregation of a core paraspeckle protein, NONO, in cellular and mouse models of FUS pathology as well as in the spinal cord of ALS-FUS patients [55]. Since both FUS and NONO are required to build paraspeckles, formation of these RNA granules was expected to be disrupted in ALS-FUS. However, this assumption has not been tested experimentally.

In the current study, using novel cell lines expressing endogenous mutant FUS, patient fibroblasts and human post-mortem tissue, we have identified excessive assembly of dysfunctional paraspeckles as a novel nuclear pathology caused by *FUS* mutations.

## Materials and methods

### Generation of cell lines with targeted modification of the FUS gene

Guide RNA target sequences within the *FUS* gene were identified using Feng Zhang lab’s Target Finder (https://zlab.bio/guide-design-resources). Respective forward and reverse oligonucleotides were annealed and cloned into pX330-U6-Chimeric_BB-CBh-hSpCas9 (pX330) vector (Addgene) according to the previously described protocol [13]. SH-SY5Y human neuroblastoma cells were split onto a 35 mm dish at 50–60% confluency one day prior to transfection. Equal amounts of plasmids (3.6 μg each) carrying upstream and downstream gRNA target sequence (or one plasmid for FUS knockout) were delivered into cells by calcium phosphate transfection. After 24 h, cells were resuspended at ~ 10–20 cells/ml and plated onto 10 cm dishes. Single-cell derived clones were expanded and screened by immunofluorescence and PCR. For sequencing of the edited portion of *FUS* gene, the PCR product corresponding to the edited allele was cloned into Zero Blunt® TOPO® vector (Life Technologies), and at least four colonies were sequenced. Primers used for PCR screening and TOPO® cloning: ΔNLS lines: 5’-TGGGGACAGAGGTGGCTTTG-3′ and 5’-CCTTCCTGATCGGGACATCG-3′; FUS KO: 5’-ACCATTTGAGAAAGGCACGCT-3′ and 5’-CACGGATTAGGACACTTCCAGT-3′.

### Cell line maintenance, differentiation, transfection and treatments

SH-SY5Y neuroblastoma cells were maintained in 1:1 mixture of Dulbecco’s Modified Eagle’s Medium and F12 medium supplemented with 10% fetal bovine serum (FBS), penicillin-streptomycin and glutamine (all Invitrogen). Cells were transfected in 24-well plates with plasmid DNA (200 ng/well), poly(I:C) (Sigma, 250 ng/well) or siRNA (AllStars Negative Control from Qiagen or NEAT1 Silencer Select®, n272456 from Life Technologies) using Lipofectamine2000. Final concentrations of MG132 and sodium arsenite (both Sigma) were 1 μM and 0.05 mM, respectively. Cells were treated with actinomycin D for 3 h to induce nucleolar caps. Plasmids for expression of GFP-tagged FUS variants are described elsewhere [54]. Plasmids for NONO and SFPQ expression were prepared by inserting respective ORFs into pEGFP-C1 vector. The protocol for obtaining human fibroblasts from a control subject and a patient with FUS P525L mutation [9, 36] was approved by the University of Palermo Review Board (prot.07/2017). Human fibroblasts were cultured under the same conditions as SH-SY5Y cells. Primary murine hippocampal cultures were prepared and transfected as described [30].

### Immunocytochemistry, RNA-FISH and proximity ligation assay (PLA) on cultured cells

Cells were fixed on coverslips with 4% paraformaldehyde for 15 min, washed with 1xPBS and permeabilized in cold methanol (or 70% ethanol in case of RNA-FISH). For immunostaining, coverslips were incubated with primary antibodies diluted in blocking solution (5% goat serum/in 0.1% Tween 20/1xPBS) for 1 h at RT or at 4 °C overnight. Secondary Alexa488- or Alexa546-conjugated antibody was added for 1 h at RT. For RNA-FISH, commercially available NEAT1 probes (Stellaris® Quasar® 570-labelled against 5′ or middle segment of human NEAT1, Biosearch Technologies) and Cy5-labelled oligo(dT)30 probe (for polyA+ RNA detection, Sigma) were used as per standard Biosearch Technologies protocol. For colocalisation studies of NEAT1 and NONO, RNA-FISH was followed by 30 min incubation in anti-NONO antibody and Alexa488-conjugated secondary antibody. PLA was performed using Duolink® In Situ Orange Starter Kit Mouse/Rabbit (DUO92102, Sigma) using anti-FUS (mouse monoclonal, Santa Cruz, sc-47711) antibody in combination with rabbit anti-NONO or SFPQ (A301-322A, Bethyl) antibody. To detect FUS and NONO interaction in paraspeckles, 1:10,000 antibody dilutions were used. Fluorescent images were captured using BX61 microscope equipped with F-View II camera and processed using CellF software (all Olympus). Quantification of paraspeckle numbers/NEAT1-positive area and PLA results was performed using ‘Analyze particles’ tool of ImageJ software. Images were prepared using Photoshop CS3 or PowerPoint 2010 software.

### RNA analysis

Analysis NEAT1_2 and MALAT1 extractability was performed as described [11]. Briefly, one set of samples lysed in QIAzol (Qiagen) was heated at 55 °C for 10 min and the second set of samples prepared in parallel was left at room temperature. RNA was extracted from both sets as per standard QIAzol protocol. Fold extraction of NEAT1_2 or MALAT1 was calculated as a ratio between levels of these RNAs, measured by qRT-PCR, in heated versus non-heated samples. For obtaining nuclear soluble extract (SNE), a protocol by Werner and Ruthenberg was followed [68]. For standard gene expression and miRNA analysis by qRT-PCR, total RNA was extracted from cells using QIAzol with a heating step (55 °C for 10 min). First-strand cDNA synthesis was performed using random primers (or oligo(dT) primers for NEAT1_1 analysis in SNE) and Superscript IV (Invitrogen) or miScript II RT (Qiagen). Quantitative RT-PCR was performed as described [30]; to measure miRNA levels, forward miRNA-specific primer was used in combination with the universal reverse primer (unimiR). All primer sequences are given in Additional file 1: Table S1. For RNA-Seq, total RNA was extracted using PureLink total RNA extraction kit (Life Technologies) and possible DNA contamination was removed using RNase free DNase kit (Qiagen). RNA-Seq analysis was performed at School of Biosciences Genomics Research Hub. Libraries were prepared using the TruSeq stranded mRNA kit (Illumina) and single-end sequencing was performed on Illumina NextSeq500 (read length: 75 bp; coverage ~ 20 million reads/sample). Reads were aligned to the human reference genome (GRCh38) using STAR [16], and FPKM values were obtained using DESeq2 [38]. Reads were viewed in the IGV browser [62].

### Protein analysis

Nuclear-cytoplasmic fractionation was performed according to a published protocol (REAP) [59]. Total cell lysates and cytoplasmic fractions were prepared for Western blot by adding 2xLaemmli buffer followed by denaturation at 100 °C for 5 min. SDS-PAGE and detection of proteins were carried out as described elsewhere [53]. Quantification of Western blots was done using Image J and protein levels were normalised to beta-actin.

### Primary antibodies

The following commercial primary antibodies were used: FUS full protein (rabbit polyclonal, 11,570–1-AP); FUS N-terminus (rabbit polyclonal, Abcam, ab84078; aa. 1–50); FUS C-terminus (Bethyl, A300-294A; aa. 500–526); p54nrb/NONO (rabbit polyclonal C-terminal, Sigma); SFPQ (rabbit monoclonal, ab177149, Abcam; rabbit polyclonal, A301-322A, Bethyl); beta-actin (mouse monoclonal, A5441, Sigma). Antibodies were used at 1:500–1:1000 dilution for all applications unless stated otherwise.

### Analysis of human tissue samples

Human spinal cord paraffin sections from clinically and histopathologically characterised ALS cases and neurologically healthy individuals were obtained from the MRC London Neurodegenerative Diseases Brain Bank (Institute of Psychiatry, Kings College, London) and Sheffield Brain Tissue Bank. Consent was obtained from all subjects for autopsy, histopathological assessment and research in accordance with local and national Ethics Committee approved donation. Human spinal cord sections for immunohistochemistry were 7 μm thick. Immediately after antigen retrieval in citrate buffer, slides were washed several times in 2xSSC prepared with DEPC-treated water. Slides were incubated with NEAT1 (5′ segment) Stellaris® probe diluted in hybridisation buffer (10% formamide/2xSSC; 5 μl probe in 200 μl buffer per slide under a 24 × 60 mm coverslip) in a humidified chamber at 37 °C overnight. Nuclei were stained with DAPI. Paraspeckles were analysed using the same microscope and camera as above (× 100 magnification). For RNAscope® ISH analysis, Hs-NEAT1-long (411541) probe (Advanced Cell Diagnostics) was used according to manufacturer’s instructions. SFPQ immunohistochemistry on spinal cord sections was performed using SFPQ IHC-00304 antibody (Bethyl) as described earlier [55].

### Quantifications and statistics

N in all cases indicates the number of biological replicates. On all graphs, error bars represent SEM. Statistical analysis was performed using GraphPad Prism 6 software. Mean values of biological replicates were compared using appropriate tests (stated in figure legends). Significance levels are indicated with asterisks (*p < 0.05, **p < 0.01, ***p < 0.001, ****p < 0.0001).

## Results

**Generation and characterisation of cell lines expressing endogenous mutant FUS.**

The requirement of FUS for paraspeckle assembly limits the use of cell models with FUS overexpression or knockdown. Moreover, patient derived pluripotent cells and neurons differentiated from these cells were also unsuitable for this study since both of these cell types lack paraspeckles [8, 43]. Therefore we chose to generate human neuroblastoma SH-SY5Y cell lines expressing endogenous mutant FUS.

**Fig. 1:**
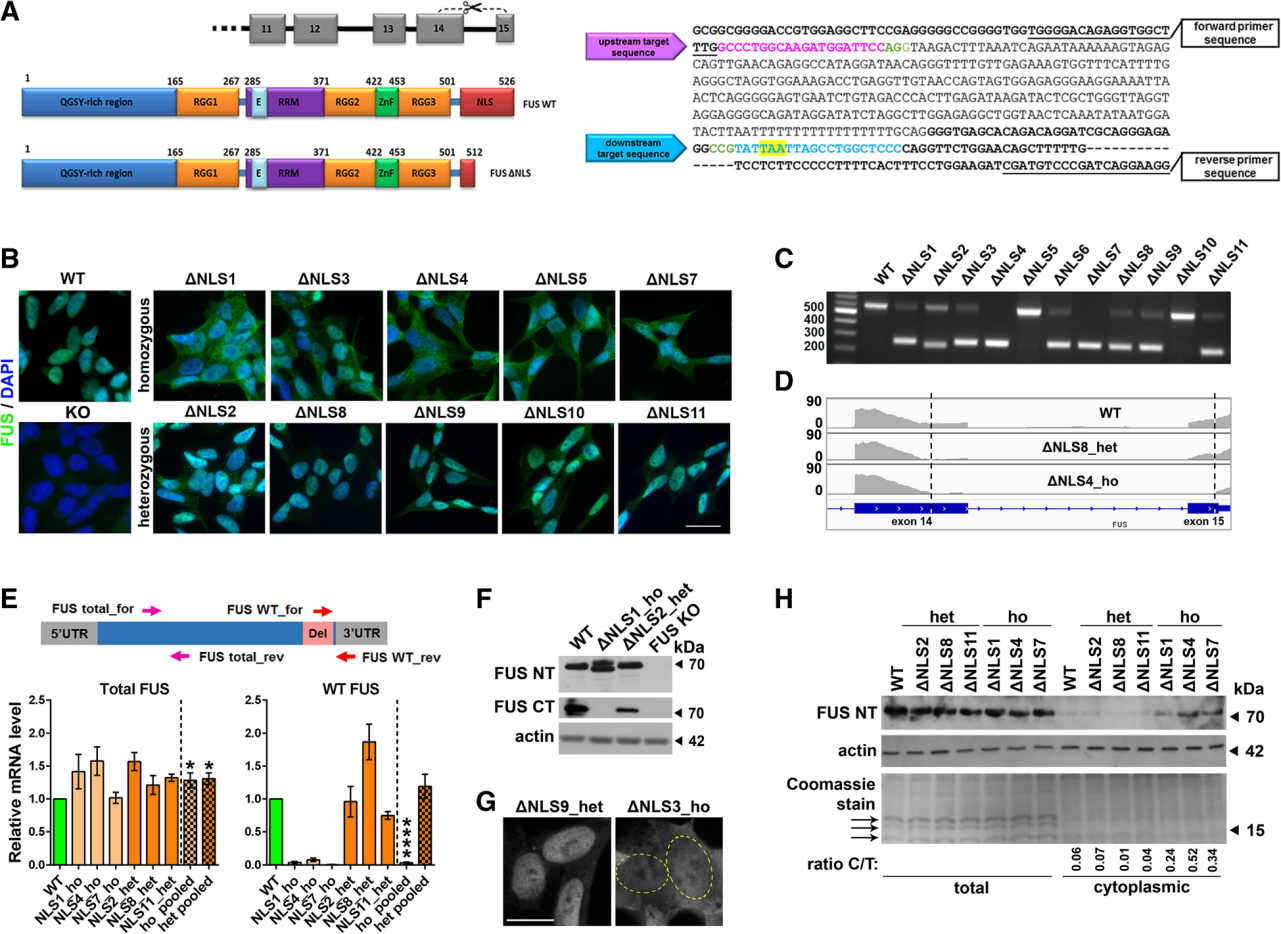
Generation and characterisation of SH-SY5Y cell lines with targeted modifications of the endogenous *FUS* gene. **a** Structures of the *FUS* gene and FUS protein together with the positions of CRISPR/Cas9 target sites chosen to delete the NLS-encoding fragment. PAM sequences are in green, stop codon is highlighted in yellow and exons are in bold. **b** Subcellular distribution of FUS protein in FUS ΔNLS and FUS knockout (KO) clones detected with N-terminal FUS antibody. **c** PCR genotyping of FUS ΔNLS clones. PCR with primers flanking the fragment to be deleted (underlined in A) yields 595 and 265 bp fragments for WT and edited *FUS* alleles, respectively. **d** RNA-Seq reads for exons 14 and 15 of the *FUS* gene in WT cells as well as heterozygous and homozygous FUS ΔNLS lines. Dashed lines indicate the deletion. **e** Analysis of FUS mRNA levels by qRT-PCR in FUS ΔNLS lines. Diagram shows positions of primers for measuring total and WT mRNA (not drawn to scale, *del* denotes the deleted region). Average values for three heterozygous (“het pooled”) and three homozygous (“ho pooled”) lines are also shown. *N* = 4–6, *p < 0.05, ****p < 0.0001 (one-way ANOVA). **f** Western blot analysis of FUS in FUS ΔNLS and FUS KO lines using antibodies recognising its N-terminal (aa.1–50) or C-terminal (aa.500–526) segments. Note that mutant FUS possesses a FUS-unrelated C-terminal amino acid stretch both in ΔNLS1_ho and ΔNLS2_het lines causing slower migration of the mutant protein (for protein sequences see Additional file 1: Figure S1A). **g** FUS distribution in representative heterozygous and homozygous FUS ΔNLS lines. Nuclei border in homozygous cells is indicated with a dashed line. **h** FUS levels in total lysates and cytoplasmic fraction from WT and FUS ΔNLS lines. Ratio C/T, ratio cytoplasmic to total FUS levels. Note absence of histones (arrows) in the cytoplasmic fraction. Scale bars, 10 μm

The majority of known ALS-FUS linked mutations disrupt the function of the NLS at the FUS C-terminus; clinically more severe variants are associated with NLS deletions [14, 34]. To mimic genetic alterations typical for the majority of ALS-FUS cases, cell lines with the deletion of genomic sequences encoding the 12 C-terminal amino acids of FUS were produced using CRISPR/Cas9 editing. For that, upstream and downstream guide RNA target sequences in exons 14 and 15 of the *FUS* gene respectively were chosen (Fig. 1a). Single-cell derived clones were screened by FUS immunostaining, and cell lines from 11 clones showing cytoplasmic redistribution of FUS were established (Fig. 1b). PCR analysis and sequencing of the edited portion of the *FUS* gene showed that 6 clones were homozygous and 5 clones were heterozygous for FUS NLS deletion (Fig. 1c, Additional file 1: Figure S1A). Interestingly, sequencing also revealed that some clones which appeared heterozygous for the *FUS* gene deletion by PCR (such as ΔNLS1) were in fact homozygous for FUS protein truncation; in these clones, inversion and re-insertion of the edited genomic DNA fragment occurred (Additional file 1: Figure S1A). RNA-Seq confirmed lower number of reads in the targeted gene fragment between exons 14 and 15 in the heterozygous clones and their absence in the homozygous clones (Fig. 1d). CRISPR/Cas9 was also used to obtain FUS knockout (KO) cells which lacked FUS immunoreactivity (Fig. 1b, Additional file 1: Figure S1B).

Analysis by qRT-PCR showed a small increase of FUS mRNA in FUS ΔNLS lines, consistent with the ability of FUS to autoregulate its own levels, and confirmed the absence of WT FUS mRNA in the homozygous lines (Fig. 1e). Western blot with an antibody against FUS N-terminus showed normal levels of FUS protein (Fig. 1f). As expected, an antibody specific to the extreme C-terminus of FUS (aa.500–526) detected no FUS protein in the homozygous lines (such as ΔNLS1) and its decreased levels in the heterozygous lines (such as ΔNLS2) (Fig. 1f). Western blot also confirmed the absence of detectable FUS protein in the FUS KO line (Fig. 1f).

We noticed that FUS redistribution to the cytoplasm was very modest in the heterozygous FUS ΔNLS lines. In contrast, homozygous cells displayed dramatic FUS mislocalisation, with the border between the nucleus and cytoplasm in the FUS-immunostained cells often indistinguishable (Fig. 1g). Subcellular fractionation confirmed almost normal retention of FUS in the nucleus in the heterozygous lines (Fig. 1h). This pattern is different from the predicted two-fold increase in the cytoplasmic mislocalisation in the homozygous as compared to heterozygous FUS ΔNLS lines and suggests that the presence of non-mutated, nuclear localised FUS partially protects mutant FUS from mislocalisation. Consistent with previous literature, mutant FUS was readily recruited to cytoplasmic stress granules induced by oxidative stress (Additional file 1: Figure S2).

Thus, we established cell lines with mild and severe mislocalisation of endogenous FUS to the cytoplasm suitable for the analysis of paraspeckles.

### Mutant FUS induces the accumulation of NEAT1 isoforms and excessive paraspeckle formation

We next used NEAT1 RNA-FISH to image paraspeckles in the lines generated. In our analysis, we included three homozygous and three heterozygous (hereafter ΔNLS_ho and ΔNLS_het, respectively) FUS ΔNLS lines as well as FUS KO cells.

**Fig. 2:**
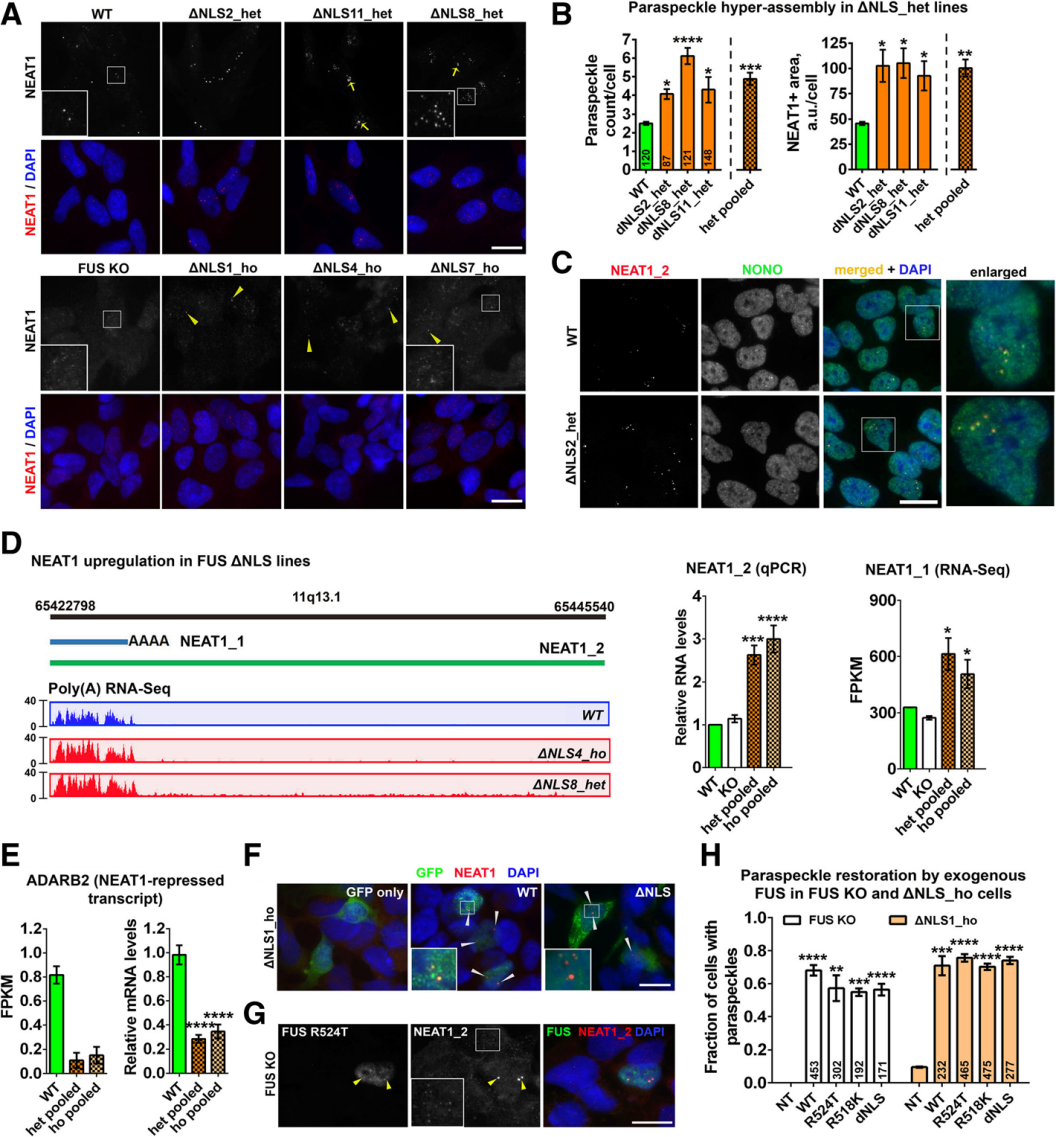
Accumulation of NEAT1 and augmented paraspeckle assembly in heterozygous FUS ΔNLS lines. **a**, **b** Cells heterozygous for the FUS NLS deletion (ΔNLS_het) have increased number of paraspeckles, whereas homozygous (ΔNLS_ho) and FUS knockout (KO) lines are almost devoid of paraspeckles. Arrows indicate clusters of paraspeckles in ΔNLS_het lines and arrowheads – residual paraspeckles in ΔNLS_ho lines (**a**). The number of NEAT1-positive foci and their area were quantified for ΔNLS_het lines (**b**). *p < 0.05, **p < 0.01, ***p < 0.001, ****p < 0.0001 (one-way ANOVA with Holm-Sidak test). **c** Paraspeckles in ΔNLS_het cells contain both NEAT1_2 and a core paraspeckle protein NONO. **d** NEAT1 isoforms are upregulated in FUS ΔNLS lines. Representative tracks for poly(A) capture RNA-Seq analysis of *NEAT1* gene in a heterozygous (ΔNLS8_het) and a homozygous (ΔNLS4_ho) lines are shown. NEAT1_1 levels were measured by RNA-Seq and NEAT1_2 levels – by qRT-PCR. *N* = 4 per line. *p < 0.05, ***p < 0.001, ****p < 0.0001 (one-way ANOVA). **e** A NEAT1-repressed transcript ADARB2 is downregulated in FUS ΔNLS lines. ADARB2 mRNA levels were measured by RNA-Seq (left) and qRT-PCR (right). *N* = 3 per line. ****p < 0.0001 (one-way ANOVA with Dunnett’s test). **f**-**h** Overexpression of FUS or its mutants restores paraspeckles in FUS KO and ΔNLS_ho cells. Arrowheads indicate mature paraspeckles or their clusters (**f**, **g**). Inset in g shows paraspeckle primary units in a non-transfected FUS KO cell. Bar chart shows the fraction of transfected ΔNLS1_ho and FUS KO cells with one or more paraspeckle (large NEAT1-positive dot) (**h**). **p < 0.01, ***p < 0.001, ****p < 0.0001 as compared to non-transfected (NT) cells (one-way ANOVA with Holm-Sidak test). All FUS variants were expressed as N-terminal GFP-fusions. Paraspeckles were visualised by NEAT1 RNA-FISH. Combined data for three heterozygous and three homozygous lines are referred as “het pooled” and “ho pooled”, respectively. In **b** and **h**, numbers of cells analysed are indicated within each bar. Scale bars, 10 μm

As predicted, FUS KO cells were devoid of paraspeckles (Fig. 2a). A similar phenotype was detected in ΔNLS_ho lines, consistent with significant FUS redistribution to the cytoplasm, although residual paraspeckles were present in some cells (Fig. 2a, arrowheads). FUS is known to act as a molecular ‘glue’ to stick individual NEAT1 RNP complexes together, to form mature paraspeckles [70]. In accord with this, in FUS KO and ΔNLS_ho cells, we observed multiple smaller NEAT1-positive dots likely corresponding to NEAT1 RNP complexes - paraspeckle “primary units” (Fig. 2a, bottom panel insets). What was surprising about our data however, was that ΔNLS_het lines displayed apparently enhanced paraspeckle formation further confirmed by automated quantification of paraspeckle numbers (Fig. 2a, b). In fact, these counts may be an underestimation as paraspeckles often form clusters counted as single foci, especially in ΔNLS_het cells (Fig. 2a, arrows). We additionally measured the cumulative area of all NEAT1-positive foci per nucleus, which also showed ~ 2-fold increase across ΔNLS_het lines (Fig. 2b). Since paraspeckles are currently defined as structures containing both NEAT1_2 and an essential paraspeckle protein [42], we used double-labeling that confirmed the presence of NONO in NEAT1_2 positive dots in ΔNLS_het lines (Fig. 2c). One of the distinctive characteristics of paraspeckle proteins is their ability to redistribute to nucleolar caps when transcription is inhibited [42, 52], and mutant FUS preserved this property (Additional file 1: Figure S3A).

Paraspeckle assembly is directly correlated with the expression of the longer NEAT1 isoform, NEAT1_2, whereas NEAT1_1, although recruited to paraspeckles, is not required for their integrity [35]. NEAT1_2 was recently reported to be “semi-extractable” meaning that heating or shearing steps are required to efficiently extract it by conventional AGPC-based methods [11]. In order to measure NEAT1_2 levels accurately, we included a heating step during RNA extraction with QIAzol. NEAT1_2, quantified by qRT-PCR, was upregulated in ΔNLS_het lines thus providing grounds for the enhanced paraspeckle assembly; however, it was similarly upregulated in ΔNLS_ho lines (Fig. 2d). NEAT1_1 completely overlaps with NEAT1_2 in its 5′ end and cannot be measured separately by qRT-PCR in total RNA samples. NEAT1_1 but not NEAT1_2 is polyadenylated. RNA-Seq analysis of poly(A)-captured RNA which only detects NEAT1_1 showed that this isoform was also significantly elevated in FUS ΔNLS lines (Fig. 2d). SFPQ and NONO are known to regulate NEAT1_2 levels and hence paraspeckle formation [42]. However, mRNA and protein levels as well as distribution of both proteins were similar in WT and FUS ΔNLS lines (Additional file 1: Figure S3B-D). Interestingly, FUS KO cells, which lack paraspeckles, displayed normal NEAT1 levels (Fig. 2d), suggesting that NEAT1 accumulation was caused by the presence of mutant FUS and not by compensatory NEAT1 upregulation in response to paraspeckle disruption. Consistent with the finding that NEAT1 is accumulated in FUS ΔNLS lines, the NEAT1-repressed mRNA ADARB2 [24] was found to be dramatically downregulated in these cells (Fig. 2e), while NEAT1 knockdown was able to elevate ADARB2 both in WT and FUS ΔNLS cells (Additional file 1: Figure S3E).

FUS itself does not stabilise NEAT1_2 and instead is involved in paraspeckle maturation downstream of NEAT1_2 synthesis [42, 70]. We next investigated whether exogenously expressed mutant FUS could restore paraspeckle assembly in FUS KO and ΔNLS_ho lines. Cells were transfected with plasmids to express GFP-tagged FUS WT, FUS ΔNLS (predominantly cytoplasmic), and ALS-linked FUS mutants R524T and R518K (predominantly nuclear) [54]. Overexpression of all FUS variants led to the appearance of bright NEAT1-positive foci in the majority of FUS KO and ΔNLS1_ho cells (Fig. 2f-h), which coincided with the disappearance of paraspeckle precursors (Fig. 2g). There were no significant differences between FUS variants in their ability to nucleate paraspeckles (Fig. 2h) – despite the fact that in cells expressing GFP-tagged FUS ΔNLS, the level of ectopic protein in the nucleus was much lower than in cells expressing other FUS variants (Fig. 2f). This suggests that a certain threshold for nuclear FUS level is required for paraspeckle assembly and that FUS mutants can maintain the formation of visible paraspeckles.

To summarise, the presence of endogenous levels of mutant FUS is accompanied by NEAT1 upregulation. This leads to increased paraspeckle numbers in cells with sufficient nuclear levels of FUS. However, more pronounced FUS mislocalisation, seen in cells expressing two mutant copies of FUS, disrupts paraspeckles.

### Mutant FUS is deficient in maintaining the integrity and functionality of paraspeckles

**Fig. 3:**
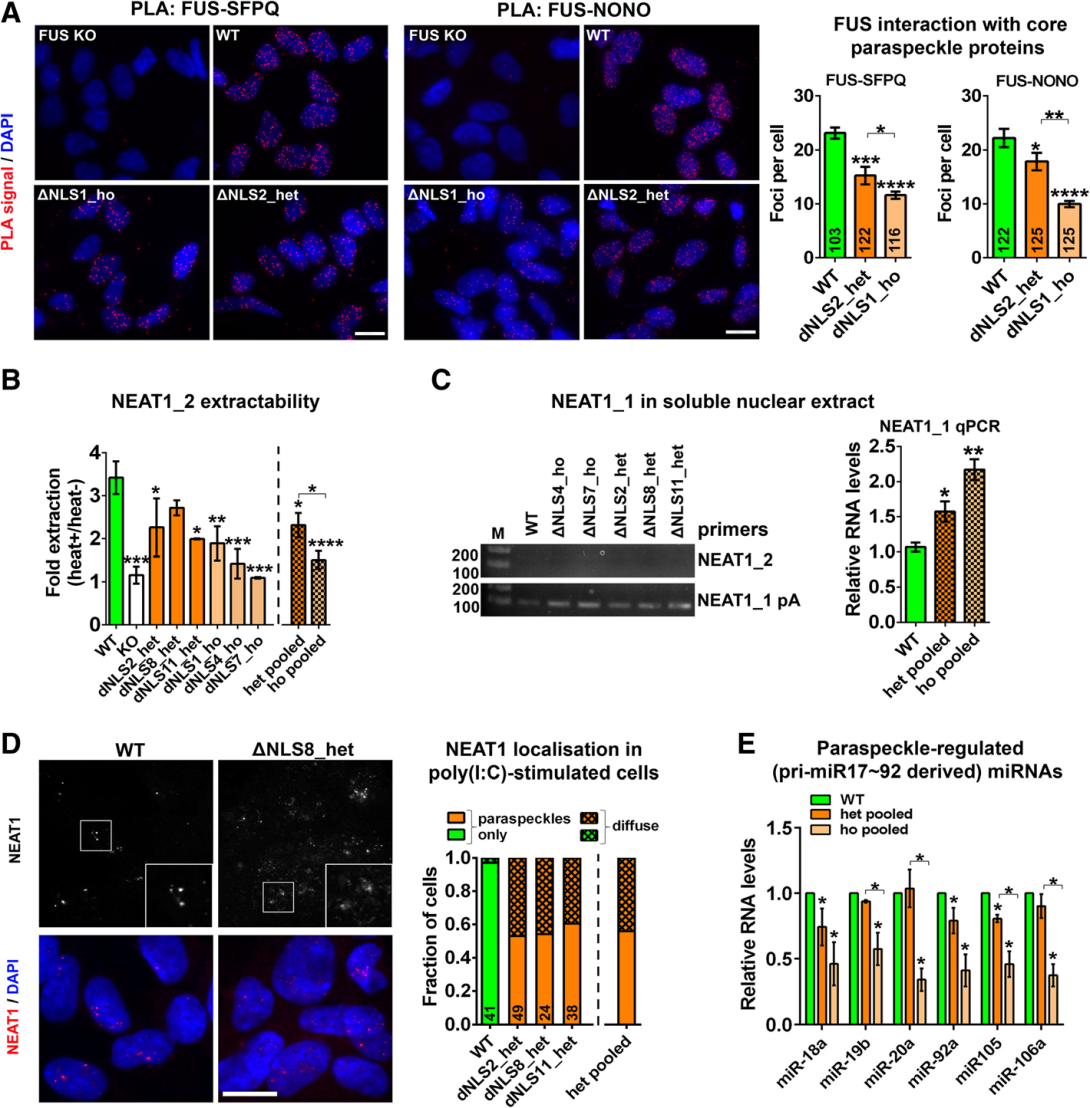
Structural and functional deficiency of paraspeckles in FUS ΔNLS lines. **a** Interaction of FUS with SFPQ and NONO is reduced in FUS ΔNLS lines as revealed by proximity ligation assay (PLA). PLA was performed in a heterozygous (ΔNLS2_het) and a homozygous (ΔNLS1_ho) lines; FUS KO cells were used as a negative control. Representative images and quantification (number of single interactions (dots) per cell (foci per cell)) are shown. *p < 0.05, **p < 0.01, ***p < 0.001, ****p < 0.0001 (one-way ANOVA with Holm-Sidak test). **b** Extractability of NEAT1_2 is increased in FUS ΔNLS lines. NEAT1_2 extractability was analysed by determining its levels in QIAzol-lysed heated versus non-heated samples (“fold extraction”) by qRT-PCR. Note near-complete NEAT1_2 extractability in FUS KO cells (fold extraction ~ 1). See also Additional file 1: Figure S4B. *N* = 3 per line. *p < 0.05, **p < 0.01, ***p < 0.001, ****p < 0.0001 (one-way ANOVA). **c** NEAT1_1 accumulates in soluble nuclear extract (SNE) in FUS ΔNLS lines. Left, representative PCR (non-saturated conditions, 26 cycles); right, qRT-PCR analysis. A primer pair located immediately upstream NEAT1_1 polyA-tail (NEAT1 pA) was used to quantify NEAT1_1 in cDNA of polyadenylated RNA. Note that NEAT1_2 which is not polyadenylated is undetectable under these conditions. *p < 0.05, **p < 0.01 (one-way ANOVA with Holm-Sidak test). **d** NEAT1 displays diffuse distribution in poly(I:C)-stimulated ΔNLS_het lines. Cells were analysed 8 h after poly(I:C) transfection by NEAT1 RNA-FISH. Representative images and quantification of the fraction of cells with diffuse NEAT1 distribution are shown. **e** Paraspeckle-regulated miRNAs are decreased in FUS ΔNLS lines. Levels of six mature miRNAs produced from pri-miR17~92 were measured by qRT-PCR separately for heterozygous and homozygous FUS ΔNLS lines, and combined average values were plotted. *p < 0.05 (Mann-Whitney *U*-test). Combined data for three heterozygous and three homozygous lines are referred as “het pooled” and “ho pooled”, respectively. In **a** and **d**, numbers of cells analysed are indicated within each bar. Scale bars, 10 μm

Although nuclear FUS levels in ΔNLS_het lines were sufficient to maintain (enhanced) assembly of visible paraspeckles, it was not clear whether these structures preserve full integrity and functionality. Core paraspeckle proteins NONO and SFPQ interact with NEAT1_2 forming a heterodimer to nucleate paraspeckle precursors, which subsequently are bonded together by FUS. Firstly, we used proximity ligation assay (PLA) to quantify FUS interaction with NONO and SFPQ. This analysis revealed significantly decreased interaction of FUS with nuclear pools of both proteins in ΔNLS_het and ΔNLS_ho lines (Fig. 3a). PLA likely detects FUS-SFPQ/NONO interactions throughout the nucleoplasm, not only in paraspeckles. Since FUS-SFPQ/NONO complexes may have different functions in paraspeckles and outside these structures, we sought to verify that paraspeckles formed in cells of FUS ΔNLS lines are characterised by reduced interaction of FUS with the core paraspeckle proteins. We reasoned that the signal from the interactions between FUS and NONO/SFPQ would be the strongest in paraspeckles because of high local concentration of protein molecules in these compact structures. By adjusting antibody dilutions, we eventually decreased the number of FUS-NONO PLA foci down to ~ 5 per cell, which most likely correspond to clusters of paraspeckles (Additional file 1: Figure S4A). Using this protocol, we also detected significantly fewer FUS-NONO foci in ΔNLS_het cells as compared to WT cells (Additional file 1: Figure S4A). Thus, interaction of mutant FUS with core paraspeckle proteins is decreased in the nucleoplasm and in paraspeckles.

FUS has been shown to be responsible for low NEAT1_2 extractability (“semi-extractability”) [11]. Weakened interaction of FUS with SFPQ/NONO implied its reduced binding to NEAT1_2 in FUS ΔNLS lines. We tested whether NEAT1_2 extractability is altered in cells expressing mutant FUS by comparing typical RNA extraction using QIAzol with a parallel sample subjected to an additional heating step. We first confirmed that heating increases NEAT1_2 extractability ~ 3.5-fold in WT neuroblastoma cells, whereas extractability of another lncRNA, MALAT1, is not affected (Additional file 1: Figure S4B). In FUS KO cells that do not form paraspeckles, NEAT1_2 was almost fully extractable (e.g. its semi-extractability was lost - heated/non-heated ratio close to 1) (Additional file 1: Figure S4B). We further found that NEAT1_2 extractability was significantly increased not only in ΔNLS_ho lines almost lacking visible paraspeckles but also in ΔNLS_het lines, albeit to a lesser extent (Fig. 3b).

It has been reported that FUS CLIP-Seq reads map predominantly to the 5′ region of *NEAT1*, with the read density being highest in the portion of *NEAT1* gene encoding the short NEAT1_1 isoform [32]. This raises the possibility that FUS mediates the recruitment of NEAT1_1 into paraspeckles during higher-order assembly of paraspeckle precursors into mature paraspeckles, whereas the deficiency of mutant FUS in paraspeckle formation would lead to NEAT1_1 release from paraspeckles. *NEAT1* gene products were shown to be enriched ~ 10-fold in chromatin-bound fraction [68] indicating that paraspeckles are co-pelleted with chromatin. We obtained nuclear soluble extract (SNE) using this protocol [68] and prepared cDNA using oligo(dT) primer in order to amplify only polyadenylated transcripts and hence only NEAT1_1 but not NEAT1_2. Indeed, NEAT1_1 levels in SNE, as quantified by non-saturated PCR and qRT-PCR, were significantly higher in FUS ΔNLS lines as compared to WT cells (Fig. 3c), indicating abnormal release of NEAT1_1 from paraspeckles in mutant FUS expressing cells.

We speculated that compromised ability of mutant FUS to maintain paraspeckle formation might become more evident under stress conditions. To test this, we used a viral infection mimic, synthetic dsRNA poly(I:C), a pathophysiological stimulus reported to enhance NEAT1 synthesis and paraspeckle formation [26]. In ΔNLS_het lines, a significant proportion of poly(I:C)-treated cells had a diffuse NEAT1 signal, as opposed to well-defined paraspeckles in all WT cells (Fig. 3d), indicating that stress-induced paraspeckle assembly is indeed impaired in cells expressing mutant FUS. Similar results were obtained with another paraspeckle-inducing stressor, proteasome inhibitor MG132 [24] (Additional file 1: Figure S4C).

Structural deficiencies in paraspeckles revealed in FUS ΔNLS lines suggested their compromised functionality. One established function of paraspeckles is positive regulation of miRNA biogenesis; in particular, paraspeckles regulate processing of pri-miR-17~92 transcript by enhancing the Microprocessor activity [27]. We found a significant decrease in the levels of six miRNAs produced from this miRNA precursor not only in homozygous but also in heterozygous FUS ΔNLS lines (Fig. 3e).

**Fig. 4:**
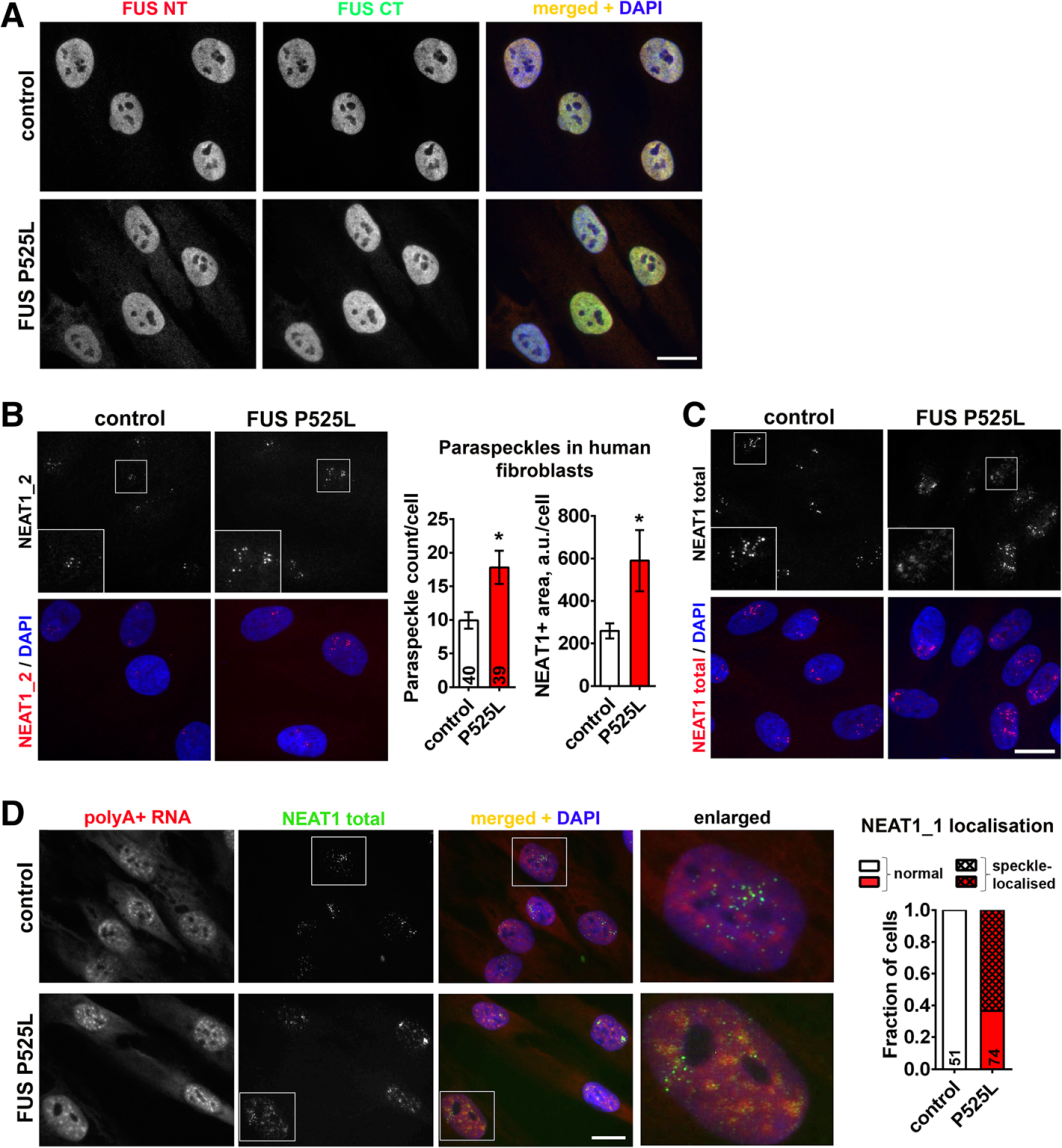
Localisation of NEAT1_1 outside paraspeckles in patient fibroblasts bearing FUS mutation. **a** FUS is predominantly nuclear in human patient fibroblasts bearing FUS P525L mutation. **b** Paraspeckle assembly is augmented in FUS P525L human fibroblasts. Paraspeckles were visualised by NEAT1_2 (3′ segment probe) RNA-FISH. *p < 0.05 (Mann-Whitney *U*-test). **c** Diffuse, non-paraspeckle distribution of NEAT1 in FUS P525L fibroblasts revealed using RNA-FISH with 5′ segment NEAT1 probe (total NEAT1). **d** NEAT1_1 is abnormally localised to nuclear speckles in FUS P525L fibroblasts. Representative images and quantification of the fraction of cells with speckle-localised NEAT1 are shown. Total NEAT1 (5′ segment probe) was used, and speckles were visualised by polyA+ RNA FISH. In **b** and **d**, numbers of cells analysed are indicated within bars. Scale bars, 10 μm

We next sought to corroborate these findings in another cellular system, human fibroblasts expressing mutant FUS. Fibroblasts are well suited for paraspeckle analysis as these cells have a large nucleus with numerous paraspeckles. In fibroblasts bearing P525L mutation FUS displayed only mild cytoplasmic mislocalisation (Fig. 4a). Consistent with data from neuroblastoma cells, paraspeckle numbers and NEAT1 positive area were increased ~ 2-fold in mutant FUS fibroblasts (Fig. 4b). Although we did not observe abnormalities in paraspeckle appearance in FUS P525L cells using NEAT1_2 probe (Fig. 4b), striking non-paraspeckle NEAT1 distribution was observed in these cells using a probe which detects both NEAT1 isoforms (total NEAT1, 5′ segment probe) (Fig. 4c). Since NEAT1_2 FISH did not produce a diffuse signal, we concluded that this abnormally localised NEAT1 corresponds to NEAT1_1. Co-localisation analysis with a polyA+ RNA, a speckle marker, showed that NEAT1_1 was mainly present on the border and/or inside speckles (Fig. 4d). This pattern is similar to NEAT1_1 ‘microspeckle’ distribution in cells lacking NEAT1_2/paraspeckles [35]. In an independent P525L fibroblast line, obtained from the same patient, but at the presymptomatic disease stage, RNA-FISH with total NEAT1 probe also detected paraspeckle disruption (Additional file 1: Figure S5). These data are in line with NEAT1_1 accumulation in nuclear soluble fraction (SNE) in FUS ΔNLS lines (Fig. 3c) and further confirm that ALS-linked mutations likely compromise the ability of FUS protein to sequester NEAT1_1 into paraspeckles.

Overall, the above results indicate that the capability of mutant FUS to maintain structural integrity and functionality of paraspeckles is impaired even in cells with minor cytoplasmic redistribution of the protein.

### Paraspeckles are formed in spinal neurons and glia of ALS-FUS patients

**Fig. 5:**
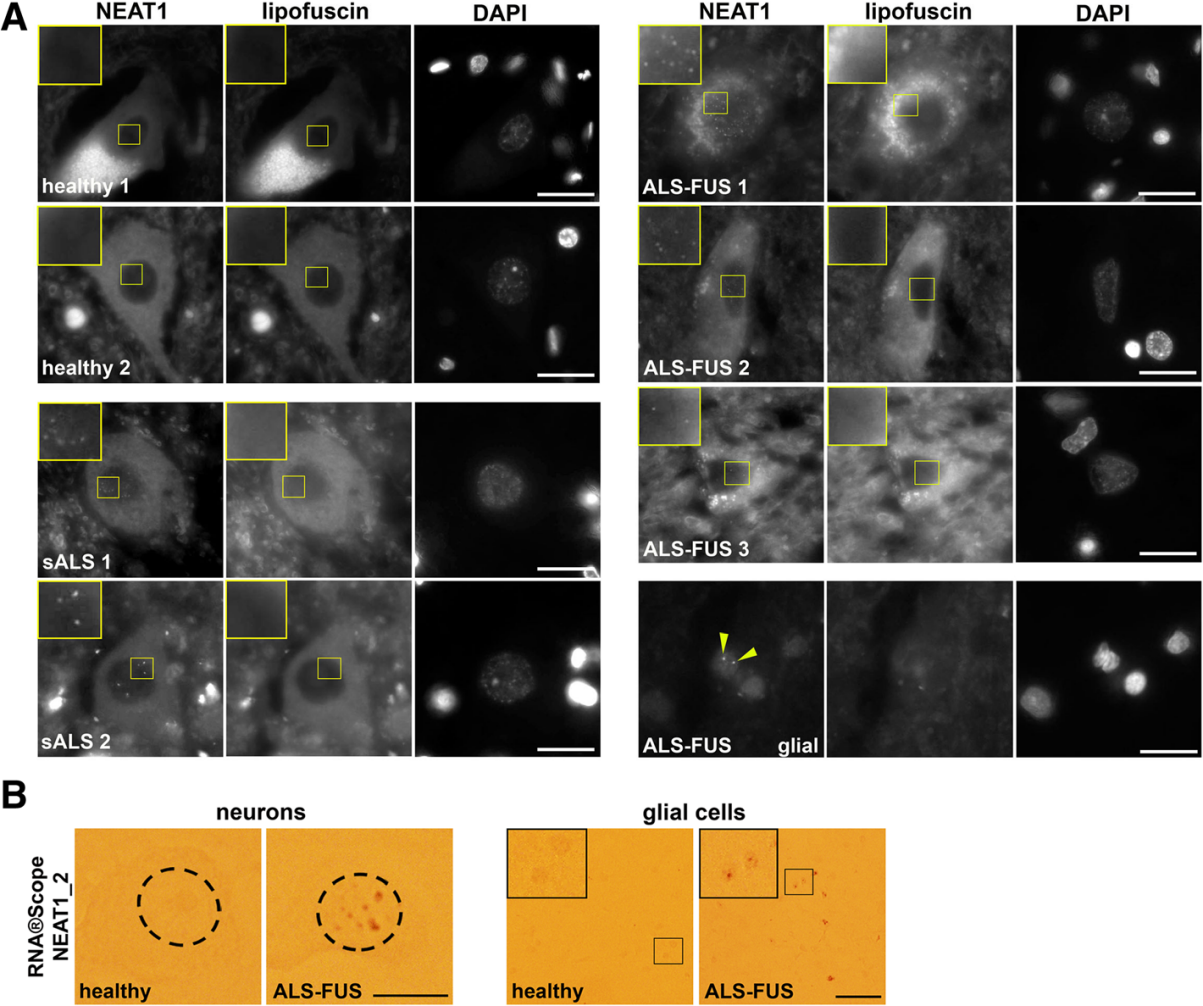
Accumulation of paraspeckles in spinal neurons and glial cells in ALS-FUS. **a** Examples of paraspeckles in spinal neurons and glial cells of ALS-FUS and sALS patients visualised using RNA-FISH with fluorescently-labelled (Quasar 570) 5′ segment NEAT1 probe. Images were taken both in the orange and green channels to distinguish between specific NEAT1 signal and autofluorescence from lipofuscin. See also Additional file 1: Table S2. Arrowheads point to paraspeckles in a glial cell. Scale bars, 10 μm. **b** Examples of paraspeckles in spinal neurons (left panels) and glial cells (right panels) in an ALS-FUS patient visualised with RNAscope® ISH using NEAT1_2 probe. Neuronal nuclei are circled. Scale bars, 10 μm (left panels) and 50 μm (right panels)

Spinal motor neurons and glial cells in sALS and fALS with TDP-43 pathology are characterised by de novo paraspeckle assembly [53]. We examined paraspeckle formation in human spinal cord sections of ALS-FUS patients by NEAT1 RNA-FISH. Three ALS-FUS cases characterised by early disease onset and, similar to the majority of ALS-FUS cases, predominantly spinal motor neuron degeneration [29], were included in the analysis (Additional file 1: Table S2); sALS cases served as a positive control. Paraspeckles were detected in all three ALS-FUS cases examined, on average being present in 27% spinal neurons (Fig. 5a, Additional file 1: Table S2), similar to what is observed in sALS and other fALS cases [53]. We also confirmed this result using RNAscope® ISH with NEAT1_2 probe (Fig. 5b). Paraspeckles were also often detected in glial cells (Fig. 5a, b). Thus, paraspeckle hyper-assembly in the spinal cord cells is a phenomenon shared by the majority of ALS cases including ALS-FUS.

In our previous study, we found that NONO is mislocalised and aggregated in ALS-FUS [55]. We examined SFPQ distribution in the same ALS-FUS cases. Although SFPQ was accumulated in the nucleus of neurons and glial cells in ALS-FUS cases, its mislocalisation or aggregation was not observed (Additional file 1: Figure S6). We also studied the behaviour of overexpressed GFP-tagged SFPQ and NONO in primary mouse neurons. In agreement with the post-mortem data, overexpressed SFPQ was confined to the nucleus, whereas NONO often mislocalised and aggregated in the cytoplasm of neurons (Additional file 1: Figure S7). Therefore nuclear SFPQ distribution is preserved in ALS-FUS allowing enhanced NEAT1 accumulation and paraspeckle assembly.

## Discussion

In the current study we provide evidence that accumulation of structurally and functionally compromised paraspeckles may serve as a novel pathomechanism in ALS-FUS. Our study reinforces the notion of enhanced paraspeckle assembly in spinal neurons and glia as a hallmark of ALS. Indeed, we show that paraspeckle formation is typical even for ALS cases with the pathology of a structural paraspeckle protein.

Paraspeckles exert anti-apoptotic activity and increase viability of cells under stressful conditions [24, 53, 67], therefore their formation in motor neurons at the early stages of pathological process in ALS may serve as a mechanism to prolong neuronal survival. However, although cells expressing mutant FUS, similar to TDP-43 depleted cells [53], are characterised by paraspeckle hyper-assembly, FUS mutations would impact on paraspeckle functionality. Disruption of paraspeckle-dependent neuroprotection may thus contribute to the particularly aggressive disease phenotype (early onset and fast progression) typical for ALS-FUS [2].

Comparison of our homozygous and heterozygous FUS ΔNLS cell lines revealed that the presence of WT FUS ameliorates mislocalisation of mutant FUS, possibly by retaining the mutant protein in the nucleus via interactions between normal and mutant FUS. It remains to be established whether nuclear retention of mutant FUS is protective or rather detrimental – e.g. by exacerbating toxic gain of function in the nucleus, including via paraspeckles. Results of previous studies of nuclear RNA granules also support gain of nuclear toxicity by mutant FUS as a disease mechanism. For example, a negative effect of mutant FUS on nuclear bodies Gems independent of its cytoplasmic mislocalisation has been demonstrated [57, 71]. In addition, FUS mutations may impact on its nuclear functions by affecting target gene expression directly [60] or via altered chromatin structure [63]. Of note, components of the chromatin remodelling complex can be recruited to paraspeckles [28]. Our results suggest that nuclear gain of function by mutant FUS may play a more important role in ALS-FUS pathogenesis than previously believed.

We found that in contrast to NONO, SFPQ does not mislocalise or aggregate in ALS-FUS, moreover, its nuclear levels are increased compared to control cases. This accumulation might play a compensatory role and serve to ameliorate the effects of NONO and FUS loss of function. Elevated SFPQ levels would also promote NEAT1 accumulation, however, since SFPQ is not significantly upregulated in FUS ΔNLS lines which nevertheless accumulate NEAT1, additional mechanisms are likely to be involved. Our transcriptomic analysis of FUS ΔNLS did not highlight any significantly dysregulated cellular pathways which could explain for NEAT1 upregulation (data not shown). It is plausible that small changes in the function of multiple pathways in mutant FUS expressing cells synergise to affect NEAT1 expression. In addition, our RNA-Seq analysis provided relatively low read coverage (~ 20 M reads/sample) and thus did not capture possible changes in the levels of low-abundance transcripts which may have impacted on NEAT1 levels. Alternatively, abnormal NEAT1 regulation can be realised at the level of posttranslational protein modifications [25].

An immediate consequence of altered structural integrity of paraspeckles in cells expressing mutant FUS is the release of NEAT1_1. NEAT1_1 is among the most abundant lncRNAs in human cells [22, 35] including those lacking paraspeckles, such as neurons. It functions to modulate transcription, including via regulation of chromatin active state [7, 35, 69]. It is highly likely that elevated levels of NEAT1_1 in neurons will cause wide-spread changes in gene expression. Recently, NEAT1_1 has been shown to interact with the p53 pathway [1, 40] and modulate neuronal excitability [5]. The latter study is especially intriguing because it suggests that elevated neuronal NEAT1_1 levels in ALS may directly contribute to their abnormal excitability [4]. Further studies are required to decipher molecular mechanisms responsible for NEAT1 upregulation and to establish whether accumulated NEAT1_1 is a significant driver of global gene expression changes in mutant FUS expressing cells.

Another important finding of our current study relevant to ALS pathogenesis is the severe repression of ADARB2 expression in mutant FUS expressing cells. ADARB2 is mainly expressed in the nervous system and was shown to be sequestered into C9ORF72 foci suggesting loss of its function in ALS-C9 [17], although possible functional consequences of this effect are yet to be addressed. ADARB2 depletion, despite mediated by a different upstream mechanism, might be a converging phenotype in ALS-FUS and ALS-C9.

Finally, our results suggest that the role of FUS in miRNA biogenesis [41] can be at least in part mediated by paraspeckles, and now it needs to be addressed to what extent pri-miRNA processing relies on the assembly of mature paraspeckles by FUS.

In conclusion, our study identifies a novel molecular phenotype driven by loss and gain of nuclear function of mutant FUS which may contribute to the disease severity in ALS-FUS.

## Additional file

Additional file 1: (DOCX 4420 kb)

## Abbreviations

(F)ISH: (Fluorescent) in situ hybridization
AGPC: Acid guanidinium thiocyanate-phenol-chloroform extraction
ALS: Amyotrophic lateral sclerosis
FPKM: Fragments per kilobase of transcript per million mapped reads
NEAT1: Nuclear Paraspeckle Assembly Transcript 1
NLS: Nuclear localization signal
poly(I:C): Polyinosinic:polycytidylic acid
RNP: Ribonucleoprotein
SNE: Soluble nuclear extract
