# Supplementary material for: ALS-linked FUS mutations confer loss and gain of function in the nucleus by promoting excessive formation of dysfunctional paraspeckles
Source: Acta Neuropathol Commun. 2019 Jan 14;7:7. doi: 10.1186/s40478-019-0658-x (PMC6330737; doi:10.1186/s40478-019-0658-x)
Supplement: Supplementary file 1 — (DOCX 4420 kb) [file 40478_2019_658_MOESM1_ESM.docx]

**
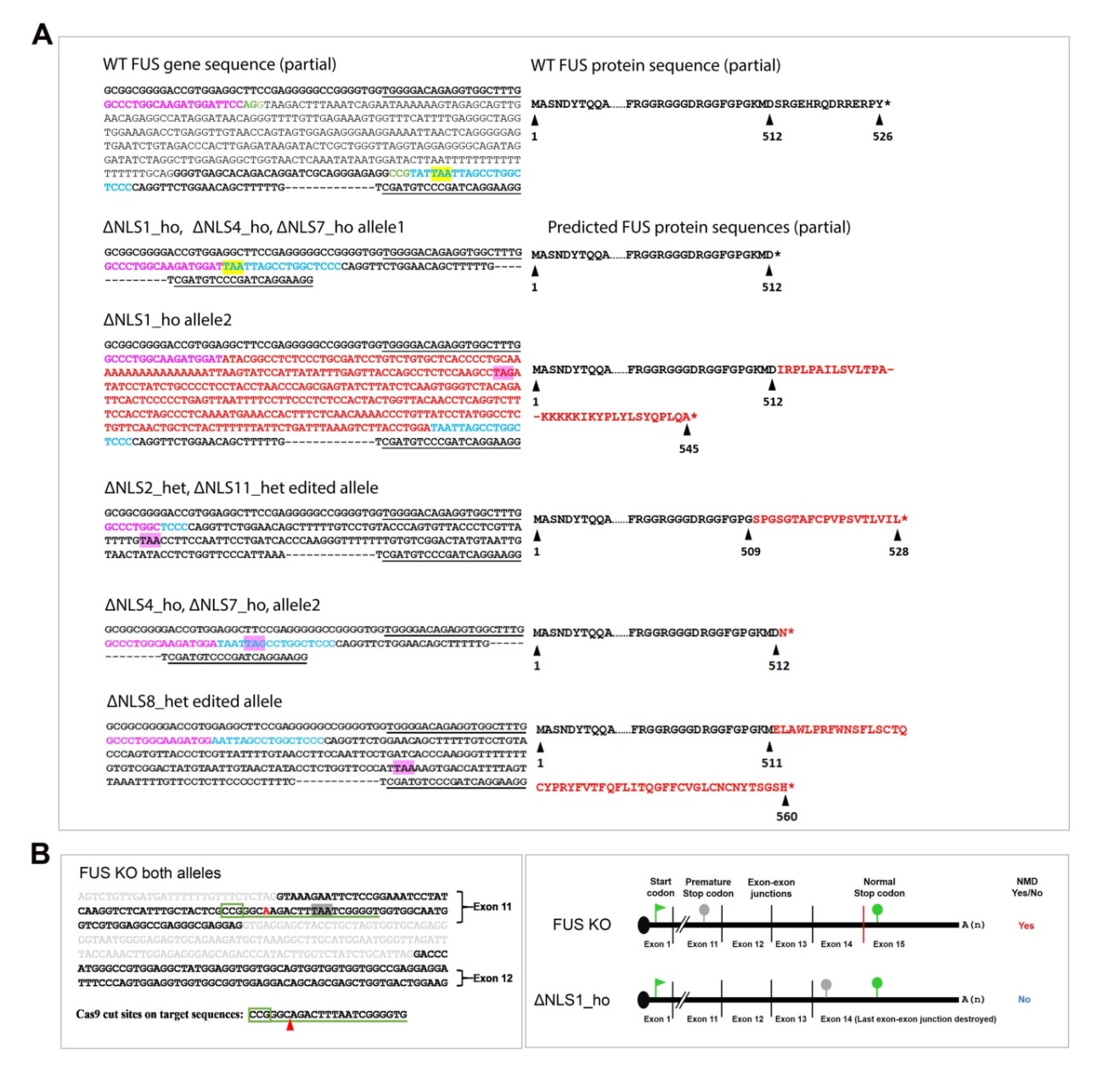
**

**Additional file 1: Figure S1. Characterisation of FUS ΔNLS and FUS KO SH-SY5Y lines.**

(A) DNA and predicted protein sequences of the NLS region in FUS ΔNLS lines used in the study. DNA fragments of the *FUS* gene were amplified, cloned into Zero Blunt TOPO vector and sequenced. Sequences were aligned to the normal *FUS* gene sequence and cut sites of Cas9 were determined. Bold letters indicate exons. PCR primers are underlined. Upstream and downstream Cas9-targeted sequences are in pink and blue respectively. PAM sequences are in green font. Stop codon is highlighted in yellow and alternative stop codons - in pink. Inverted DNA fragment is shown in red. FUS-unrelated amino acids resulting from frameshifts are given in red.

(B) Characterisation of FUS KO cells. Left panel: partial sequencing of the *FUS* gene in FUS KO cell line covering the CRISPR/Cas9 target site indicates that a premature stop codon (in grey) has been introduced in exon 11 due to scarring at the edited site (insertion of one nucleotide, red font). Right panel: diagram showing the position of a premature stop codon in the *FUS* gene of FUS KO line. A stop codon in this position should trigger mRNA degradation by nonsense-mediated mRNA decay (NMD). An example of editing outcome when NMD is not triggered (such as in ΔNLS1_ho clone) is also given.

**
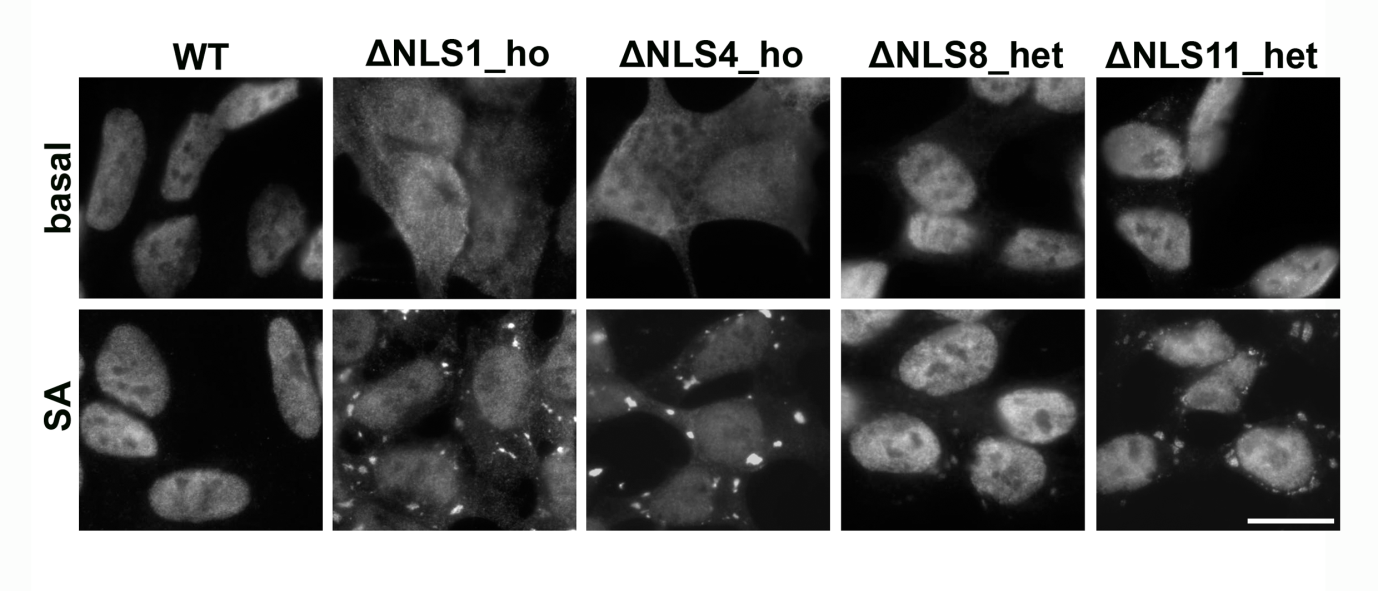
Additional file 1: Figure S2.** Mutant FUS is recruited to sodium-arsenite induced stress granules in FUS ΔNLS cell lines. Cells were treated with sodium arsenite for 1 h. Scale bar, 10 µm.


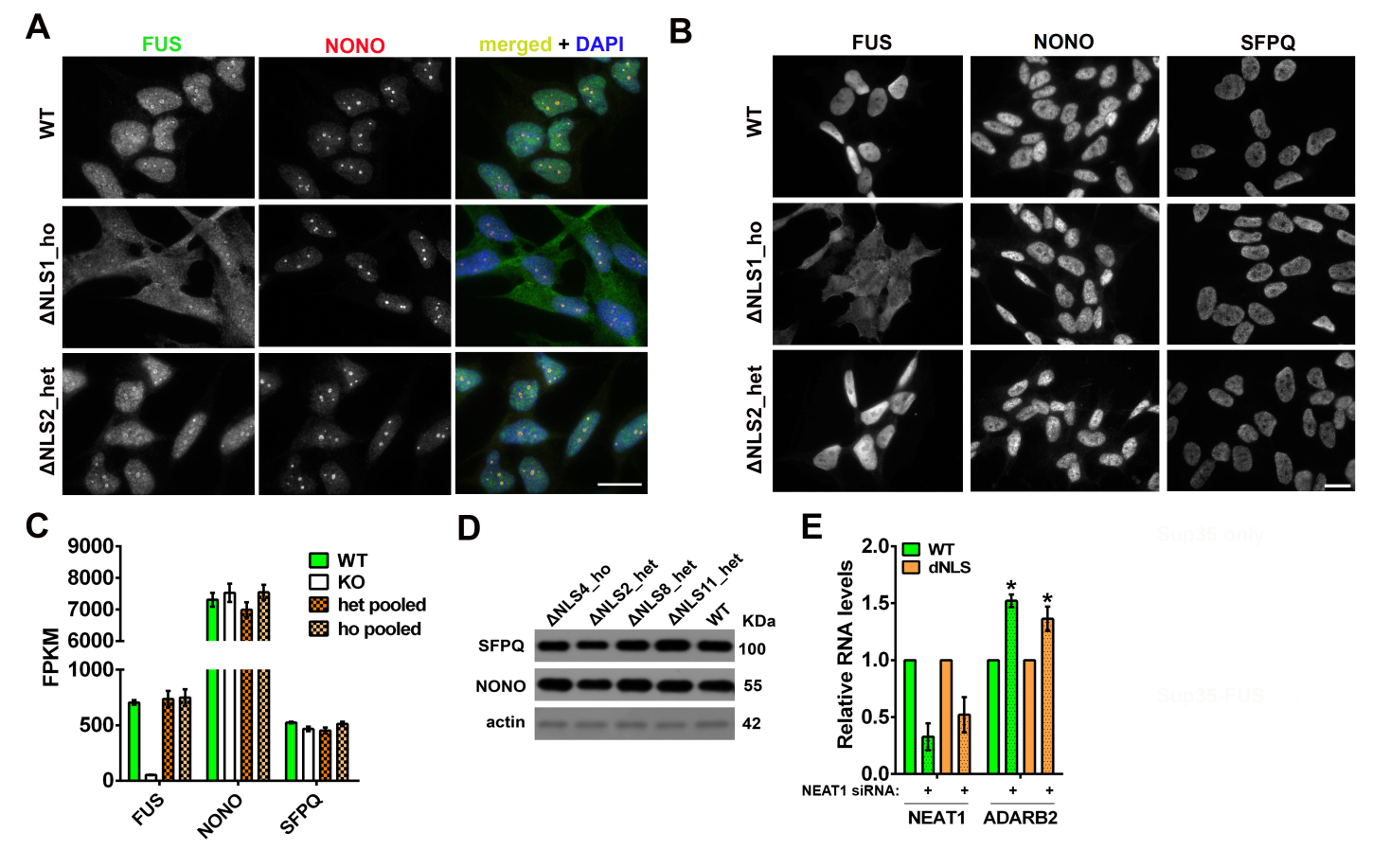


**Additional file 1: Figure S3. Paraspeckle proteins in FUS ΔNLS lines.**

(A) Mutant FUS retains its ability to be recruited to nucleolar caps when transcription is inhibited. Cells were treated with actinomycin D for 3 h and nucleolar caps were visualised with anti-NONO staining. Scale bar, 10 µm.

(B-D) Distribution (B) as well as mRNA (C) and protein (D) levels of core paraspeckle proteins are not significantly affected in FUS ΔNLS cells. Representative images and Western blots are shown in B and D. Data for three heterozygous and three homozygous lines were combined for the graph in C. Scale bar, 10 µm.

(E) NEAT1 siRNA-mediated knockdown upregulates ADARB2 in WT and FUS ΔNLS cells. Cells were analysed 48 h post-transfection. Data for three lines FUS ΔNLS were combined. N=3, *p<0.05 (Mann-Whitney *U*-test).


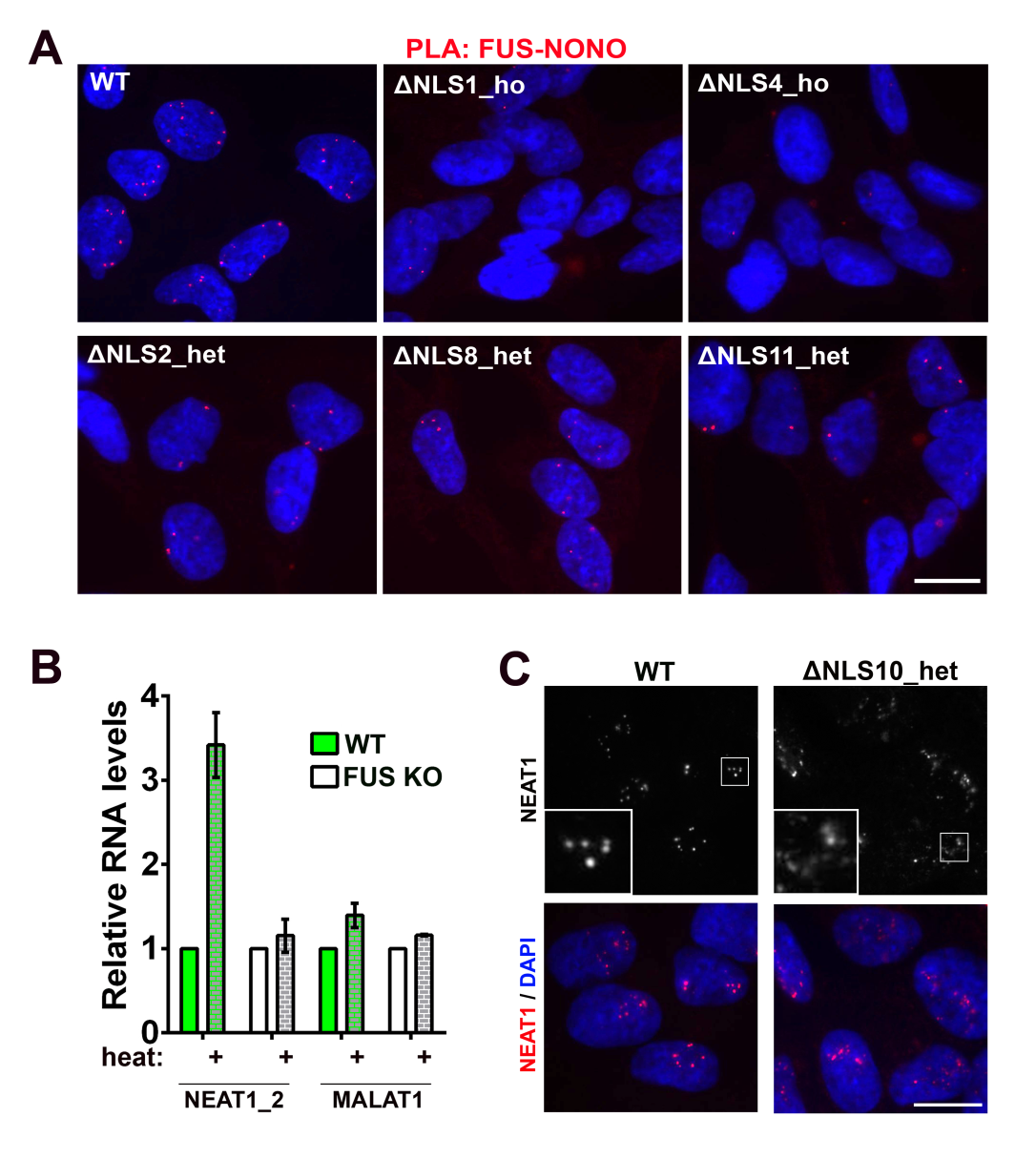


**Additional file 1: Figure S4. The effect of mutant FUS on paraspeckles in SH-SY5Y lines.**

(A) Modified proximity ligation assay (PLA) detects decreased FUS-NONO interactions in paraspeckles in FUS ΔNLS lines. Cells were incubated with FUS and NONO antibodies at 1:10,000 dilution, and subsequently the regular PLA protocol was followed. Representative images of all lines are shown. Scale bar, 10 µm.

(B) NEAT1_2 but not MALAT1 is semi-extractable in WT neuroblastoma cells, and this property is lost in FUS KO cells. Analysis of semi-extractability is described in Materials and methods. NEAT1_2 and MALAT1 levels were measured by qRT-PCR.

(C) Treatment with a paraspeckle-inducing stressor, MG132, results in diffuse paraspeckles in ΔNLS_het lines. Paraspeckles were visualised with NEAT1 RNA-FISH (5’ segment probe). Representative images for WT and ΔNLS_10 lines are shown. Scale bar, 10 µm.

**
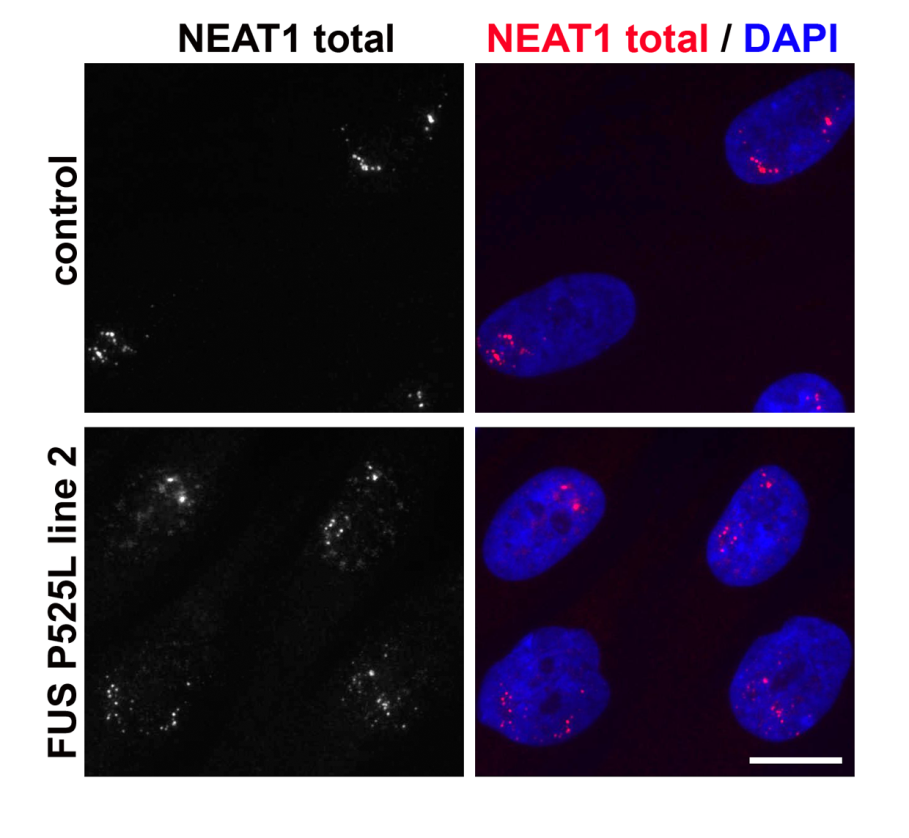
**

**Additional file 1: Figure S5. NEAT1 distribution in an additional FUS P525L line, derived from an ALS-FUS patient before disease onset.** Paraspeckles were visualised with NEAT1 RNA-FISH (5’ segment probe). Representative images are shown. Scale bar, 10 µm.

**
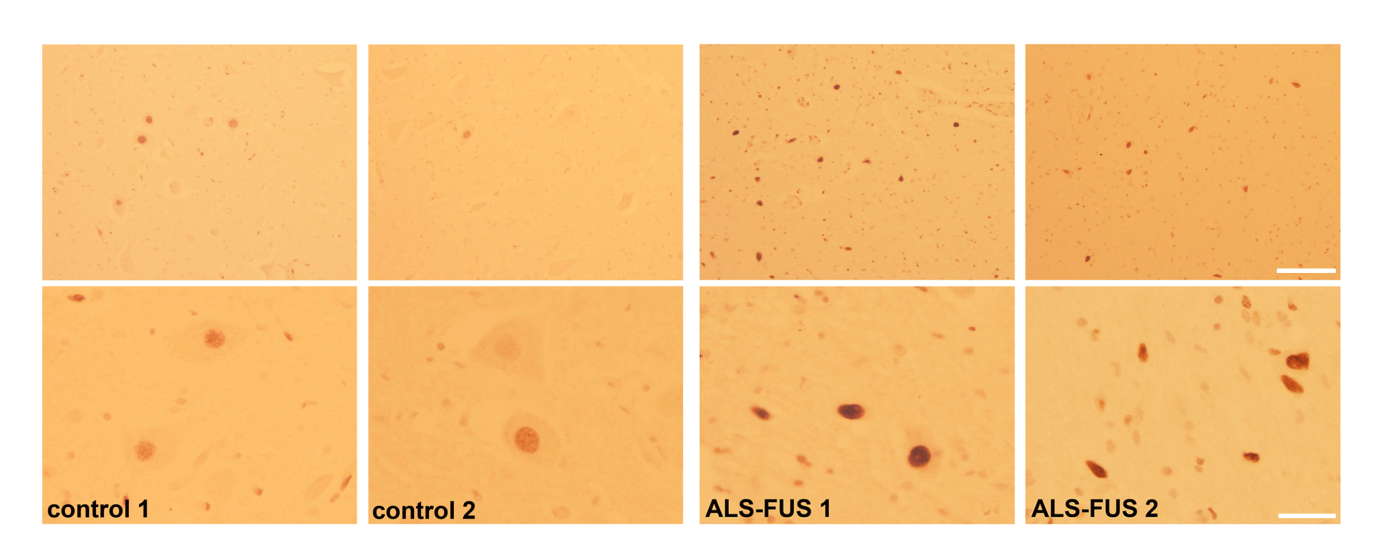
**

**Additional file 1: Figure S6. SFPQ protein accumulates in the nucleus in the spinal cord of ALS-FUS without signs of aggregation.** SFPQ immunoreactivity in the spinal cord of two ALS-FUS patients and two healthy (control) individuals. Images for ALS-FUS cases #1 and #2 from Table S2 are shown. Scale bars, 100 and 20 µm for general plane (top) and close-up (bottom) panels, respectively.


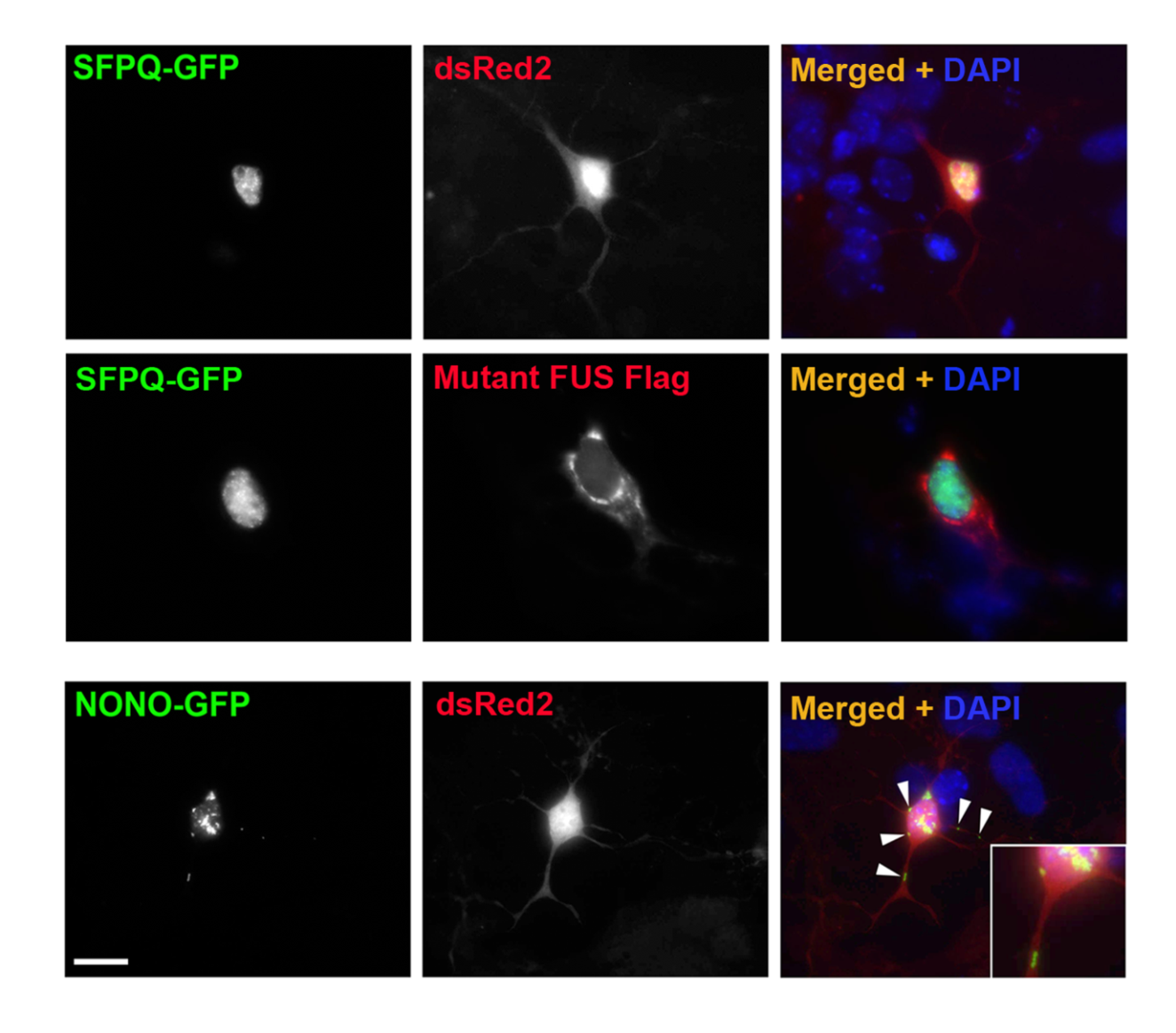
**Additional file 1: Figure S7. Distribution of overexpressed SFPQ and NONO proteins in mouse primary hippocampal neurons.** Overexpressed SFPQ preserves nuclear localisation even in the presence of mutant cytoplasmic FUS (top and middle panels), whereas overexpressed NONO can redistribute to and aggregates in the cytoplasm, even in the absence of mutant FUS (bottom panel). DIV5 mouse neurons were transfected to co-express dsRed2 or Flag-tagged FUS R522G and a GFP-tagged paraspeckle protein and analysed 24 h post-transfection. Arrowheads indicate cytoplasmic NONO aggregates. Scale bar, 10 µm.

**Additional file 1: Table S1. Primers used in the study**

| **Target** | **Forward** | **Reverse** |
| --- | --- | --- |
| GAPDH | 5’-TCGCCAGCCGAGCCA-3’ | 5’-GAGTTAAAAGCAGCCCTGGTG -3’ |
| FUS WT | 5’-GTGAGCACAGACAGGATCGC-3’ | 5’-GAGGGTAACACTGGGTACAGG-3’ |
| FUS total | 5’-GGAACTCAGTCAACTCCCCA-3’ | 5’-TACCGTAACTTCCCGAGGTG-3’ |
| NEAT1 total | 5’-CTCACAGGCAGGGGAAATGT-3’ | 5’-AACACCCACACCCCAAACAA-3’ |
| NEAT1_2 | 5’-AGAGGCTCAGAGAGGACTGTAACCTG-3’ | 5’-TGTGTGTGTAAAAGAGAGAAGTTGTGG-3’ |
| MALAT1 | 5'-GGATCCTAGACCAGCATGCC-3' | 5'- AAAGGTTACCATAAGTAAGTTCCAGAAAA-3' |
| NEAT1_1 pA | 5’-TCACGCATGTATGGGGAAGT-3’ | 5’ ACCATACAGAGCAACATACCAGT-3’ |
| ADARB2 | 5’-ATATTCGTGCGGTTAAAAGAAGGTG-3’ | 5’-ATCTCGTAGGGAGAGTGGAGTCTTG-3’ |
| miR-18a_for | 5'-CATCATCGGTAAGGTGCATC-3' | 5’-GAATCGAGCACCAGTTACGC-3’ (unimiR) |
| miR-19b_for | 5’-GCACTGACATGTGCAAATCC-3’ | unimiR |
| miR-20a_for | 5’-CGCACGACTAAAGTGCTTATAG-3’ | unimiR |
| miR-92a_for | 5’-GAGTCTATTGCACTTGTCCC-3’ | unimiR |
| miR-105_for | 5’-TCAAATGCTCAGACTCCTGTGGT-3’ | unimiR |
| miR-106a_for | 5’-AAAAGTGCTTACAGTGCAGGTAG-3’ | unimiR |

**Additional file 1: Table S2. Characteristics of ALS-FUS cases used in the study**

| **Patient No** | **Sex** | **Age at onset** | **Mutation** | **FUS pathology in spinal cord** | **References** | **Paraspeckles**  **(neurons**  **positive for paraspeckles/ total neurons analysed)** |
| --- | --- | --- | --- | --- | --- | --- |
| 1 | M | 33 | p.R521C | FUS-positive GCI | Vance et al., 2009; King et al., 2015 | 1/5 |
| 2 | F | 35 | p.R521C | FUS-positive NCI and GCI |  | 3/10 |
| 3 | F | 35 | p.R521H | FUS-positive NCI and GCI |  | 2/7 |

GCI – glial cytoplasmic inclusions

NCI – neuronal cytoplasmic inclusions
